# A Multi-species Bait for Chagas Disease Vectors

**DOI:** 10.1371/journal.pntd.0002677

**Published:** 2014-02-27

**Authors:** Theo Mota, Ana C. R. Vitta, Alicia N. Lorenzo-Figueiras, Carla P. Barezani, Carlos L. Zani, Claudio R. Lazzari, Liléia Diotaiuti, Lynne Jeffares, Björn Bohman, Marcelo G. Lorenzo

**Affiliations:** 1 Laboratório de Triatomíneos e Epidemiologia da Doença de Chagas, CPqRR-FIOCRUZ, Belo Horizonte, Brazil; 2 Departamento de Fisiologia e Biofísica, Instituto de Ciências Biológicas-UFMG, Belo Horizonte, Brazil; 3 Laboratorio de Fisiología de Insectos, IBBEA-CONICET, FCEyN, Universidad de Buenos Aires, Buenos Aires, Argentina; 4 Laboratório de Química de Produtos Naturais, CPqRR-FIOCRUZ, Belo Horizonte, Brazil; 5 Institut de Recherche sur la Biologie de l'Insecte, UMR CNRS 7261, Université François Rabelais, Tours, France; Liverpool School of Tropical Medicine, United Kingdom

## Abstract

**Background:**

Triatomine bugs are the insect vectors of *Trypanosoma cruzi*, the etiological agent of Chagas disease. These insects are known to aggregate inside shelters during daylight hours and it has been demonstrated that within shelters, the aggregation is induced by volatiles emitted from bug feces. These signals promote inter-species aggregation among most species studied, but the chemical composition is unknown.

**Methodology/Principal Findings:**

In the present work, feces from larvae of the three species were obtained and volatile compounds were identified by solid phase microextraction-gas chromatography-mass spectrometry (SPME-GC-MS). We identified five compounds, all present in feces of all of the three species: *Triatoma infestans*, *Panstrongylus megistus* and *Triatoma brasiliensis*. These substances were tested for attractivity and ability to recruit insects into shelters. Behaviorally active doses of the five substances were obtained for all three triatomine species. The bugs were significantly attracted to shelters baited with blends of 160 ng or 1.6 µg of each substance.

**Conclusions/Significance:**

Common compounds were found in the feces of vectors of Chagas disease that actively recruited insects into shelters, which suggests that this blend of compounds could be used for the development of baits for early detection of reinfestation with triatomine bugs.

## Author Summary

Chagas disease is a parasitic infection affecting approximately 12 million people, and is considered to be one of the most severe burdens for public health in Latin America. Control of the disease is based on attempted elimination of domestic populations of triatomine bugs, the insects transmitting the disease to humans, by means of insecticide spraying. Currently, vigilance programs monitoring triatomine reinfestation processes in houses are performed by manual search for bugs. Effective and sustainable new methods allowing continuous monitoring of domestic triatomine populations are required. Based on the fact that the insects hide in dark refuges that are marked by volatile signals emitted in their feces, we screened the feces of three species for volatile compounds common to these prominent vectors. The potential for these odors to promote triatomine aggregation was evaluated and we present evidence that a synthetic blend of these substances is capable of recruiting bugs into shelters, mimicking the natural pheromone. This blend may be used to develop a bait to monitor triatomine reinfestation processes in a similar manner as is used commonly for the monitoring of agricultural pests.

## Introduction

The flagellate parasite *Trypanosoma cruzi* (Chagas), the etiological agent of Chagas disease, is transmitted to humans by vectors of the subfamily Triatominae. In part as a result of the distribution of vectors, Chagas disease occurs exclusively in Latin America where it is estimated that 90 million people are at risk of transmission, while 12 million are already infected [1]. The primary vector in the Southern Cone of South America is *Triatoma infestans* [2], [3], however other species such as *Panstrongylus megistus* and *Triatoma brasiliensis*, play an important role in transmission in some regions of Brazil [4], [5]. Control of the sylvatic vector species at domiciliary ecotopes is particularly difficult as they readily invade households from wild ecotopes where they cannot be controlled with available methods [4], [5].

Triatomine bugs are obligate haematophagous insects, which feed mostly on the blood of birds and mammals. During daylight hours, these insects are usually found aggregated inside protected shelters which they leave after dusk in search of food [6]. Triatomine aggregation behavior inside shelters is well documented and is mediated by chemical signals and thigmotaxis [7]. Previously two chemical signals have been implicated in the aggregation of *T. infestans* inside the shelters: one released from feces [7]–[9] and another present in their cuticle [10], however no definitive chemical identifications were carried out.

Pheromones are substances used by organisms to transfer information between two or more members of the same species. These can be single chemical compounds or blends of several components. To date, the pheromone blend emitted by triatomine feces has not been fully described for any species. Aggregation mediated by a fecal pheromone was demonstrated in several triatomine species, including *P. megistus* and *T. brasiliensis* [11]–[16]. *T. infestans* deposit their feces in and around shelters and the volatiles emitted from fecal depositions act as chemical landmarks helping the bugs to locate their refuges [7]. It has been demonstrated that only dry feces of *T. infestans* promote aggregation, while fresh feces induces rejection [9]. Feces become attractive three hours after being deposited and attract bugs for up to 10 days [17]. *Triatoma infestans*, *Rhodnius prolixus* and *Triatoma mazzoti* showed changes in their responses to the fecal aggregation signal depending on their nutritional status [8], [17], [18]. In fact, recently fed *T. infestans* do not aggregate in response to this signal until 8–10 hours after feeding [17].

Four compounds were previously identified in polar solvent extracts of feces of *T. infestans* and *T. mazzottii* [19]; however no behavioral response was reported. Subsequently it was demonstrated that adult females and fifth instar larvae of *T. infestans* were attracted to blends of the fecal compounds 4-methylquinazoline and 2,4-dimethylquinazoline [20]. In addition it has been reported that ammonia from humidified feces attracts larvae in bioassays [21]. To the best of our knowledge no previous studies have demonstrated aggregation behavior in response to synthetic compounds.

Several authors have reported the occurrence of cross-aggregation responses to feces in diverse triatomine species [11]–[16]. The characterization of a common aggregation signal may allow for the development of chemical baits for monitoring multiple vector species.

In the present report, we first aimed to identify common and readily obtainable compounds that promote cross species aggregation in triatomines. For this, we identified volatile compounds present in the feces of *T. infestans*, *P. megistus* and *T. brasiliensis.* The behavior-modifying capacity of the volatiles common to all species was subsequently tested with larvae of each of the three species. Finally, we evaluated the potential of artificial shelters baited with a blend of these fecal volatiles for promoting the aggregation of larvae of each species. We show that a synthetic blend of substances is capable of recruiting bugs into shelters, mimicking the effects of the natural aggregation signals.

## Materials and Methods

### Insects

*Panstrongylus megistus* and *T. infestans* colonies originated from insects captured at domestic and peridomestic refuges in Minas Gerais state, while that of *T. brasiliensis* came from insects from similar ecotopes in Ceará state, Brazil. These colonies were kept for at least ten years in a rearing chamber (4.5×1.6 mt) with controlled temperature and a 12∶12 hour light:dark illumination cycle provided by artificial lights (4 fluorescent lamps, cold white light, 6400K, 40W).

As previous reports have shown that all developmental stages of triatomines make use of fecal aggregation pheromones [9], [11], we chose to use immature bugs both for our chemical and behavioral experiments because they are readily available. Triatomine larvae also have the additional benefit of not emitting alarm or sexual pheromones which could interfere in case experiments were performed with adult insects [22].

### Identification of volatile substances from samples of feces

Feces from third and fourth instar larvae of *T. infestans*, *P. megistus* and *T. brasiliensis* were collected separately in 2 ml glass vials (12×32 mm standard autosampler vials, Sigma-Aldrich) by gently pressing the abdomens of bugs with forceps. A solid phase microextraction (SPME) fiber (PDMS/DVB Stableflex, 65 µm, Supelco, Sigma-Aldrich) was exposed to the headspace of the samples for 30 min at room temperature prior to analysis by gas chromatography with mass-spectrometric detection (GC-MS Shimadzu 17A coupled to a Shimadzu 5050A). The desorption time in the injection port of the GC was 1 min. Helium was used as carrier gas (30 cm/s). GC injector and transfer line temperatures were 240°C and 250°C, respectively. The ionization energy was 70 eV. The oven program was 80°C for 1 min and 5°C/min to 240°C using a SupelcoWax-10 column (30 m×0.25 mm i.d.×0.25 µm film; Supelco, USA). Tentative identification of volatiles was based on the comparison of retention indices and mass spectra with data from the literature and a spectral library (NIST-02). All tentative identifications were confirmed by co-injections with authentic standards.

Samples of volatiles of *T. infestans* and *P. megistus* were first obtained three hours after the collection of feces (i.e., from fresh feces) and afterwards, every 24 hours during the subsequent five days (dry feces). The samples of *T. brasiliensis* were obtained during days 0 (fresh feces), 1, 3 and 5 (dry feces). In all cases, samples of feces were collected from bugs that had been fed one week earlier. The vials with samples of feces were left open in a closed room during the study, with each species sampled separately. The relative abundance of each compound was determined using the area under the peak of the chromatogram. Empty vials without feces, placed in the same room, were used as blanks.

### Behavioral responses to common compounds identified in feces

The common substances identified in samples of feces of *T. infestans*, *P. megistus* and *T. brasiliensis* were selected for behavioral tests. Standards of acetamide (Fluka), 2,3-butanediol (Supelco), acetic acid (Fluka), 3-methylbutyric acid and hexanoic acid (Sigma-Aldrich) were at least 98% pure. Individual compounds were tested with each vector species in decadic steps in dose-response assays ranging from 1 pg to 100 µg. Solutions of 2,3-butanediol, acetic acid, 3-methylbutyric acid and hexanoic acid were prepared in dichloromethane (Nanograde, Mallinckrodt) and acetamide in distilled water.

All experiments were made at 25±2°C and 65±10% R.H. using a circular glass arena (14 cm ∅) where the bottom was lined with filter paper. Two pieces of flat filter paper (1×1.5 cm) were placed on opposite halves of the arena, one impregnated with the test solution (test) and the other impregnated with pure solvent (control). These two papers were positioned 1 cm from the edge of the arena and separated by approximately 10 cm [9], [13], [14].

Two control series of tests (32 assays per series) were performed for each species. i) two pieces of clean filter paper on opposite sides of the arena, in order to test for environmental asymmetries. ii) one clean filter paper against a filter paper impregnated with dichloromethane or distilled water, in order to test for solvent effects on behavior. For each species, we performed 32 tests with each dose of the five compounds tested. In these experiments, individual insects, third instar larvae starved for 11±4 days post ecdysis, were used per test and discarded afterwards. All insects were tested during the light phase of their daily cycle, maximizing their tendency to respond to chemical signals related to shelter search. The results were analyzed by means of a binomial test.

An individual insect was placed in the center of the arena using a small inverted plastic container that avoided it to climb due to its smooth surface. After 10 minutes, the insect was released by means of a string that allowed raising the container from a distance without disturbing it. One hour later, the position of the insect was recorded. Triatomines are typically found motionless when displaying aggregation inside shelters [7] and given that we aimed to evaluate the potential role of these substances for promoting aggregation, only motionless insects were considered in our analyses.

### Testing blends of compounds in artificial shelters

**Figure 1:**
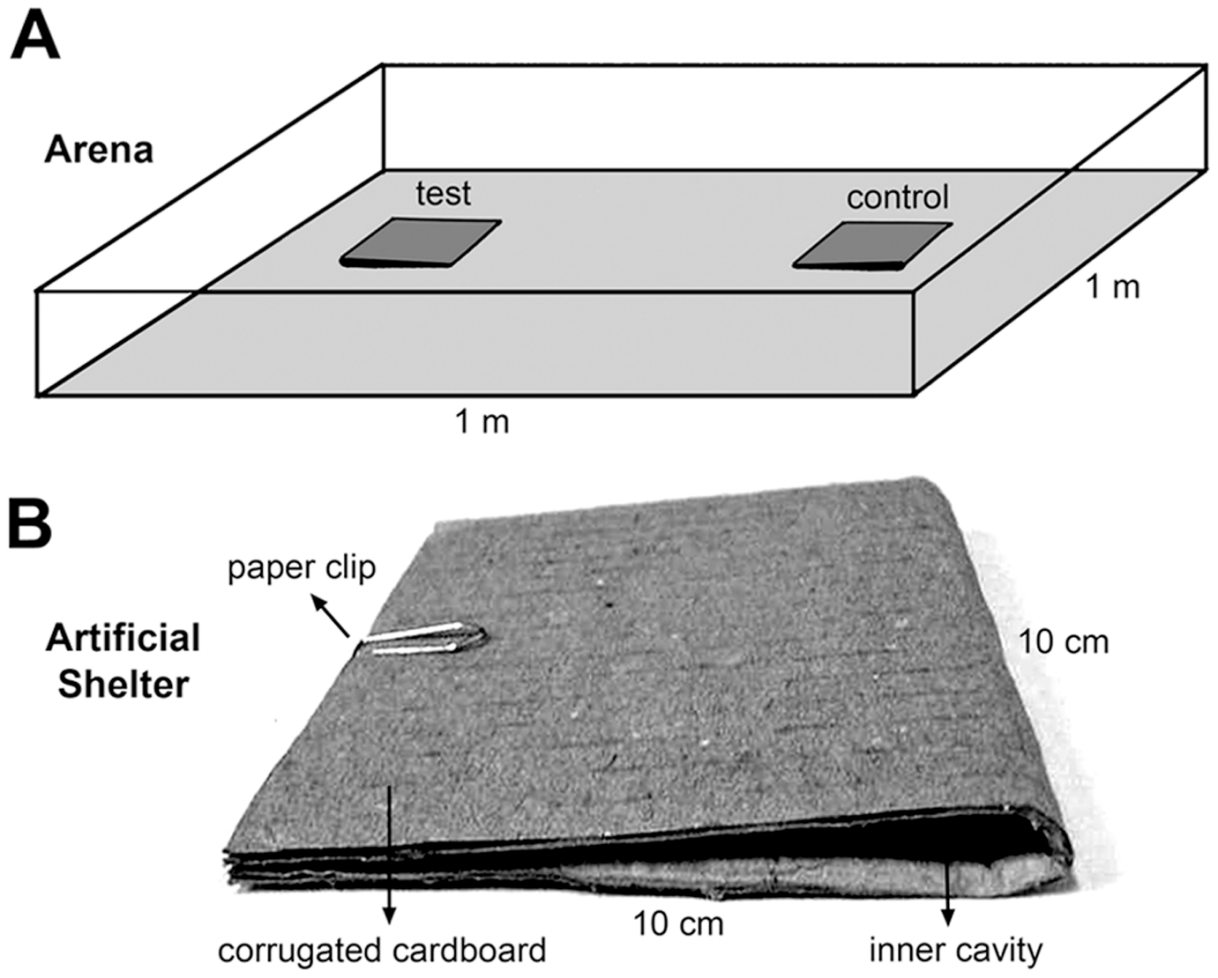
Schematic drawing showing: A- the experimental arena used in the shelter assays and, B- a picture of an artificial refuge.

A square glass arena (1 m^2^) lined with filter paper was used for these tests (Figure 1A). Two artificial shelters made from a piece of corrugated cardboard (20×10 cm), folded to generate a 10 cm^2^ shelter with two lateral slits of approximately 0.5 cm in height [7], were placed on opposite sides of the arena (Figure 1). In one of the shelters, a piece of filter paper (4×6 cm) impregnated with a blend of the five compounds was introduced, while the other shelter contained a piece of filter paper treated with solvent as control. This shelter design (Figure 1B) is proven to successfully recruit triatomine bugs [6], [7], [23], [24], which tend to enter the shelters due to their strong thigmotaxis and intense negative phototaxis [25].

Fifth instar larvae starved for 11±4 days post ecdysis were used in this experiment. A group of 30 insects was released in the center of the arena two hours before the beginning of the scotophase. The illumination of the experimental room was set to a 12∶12 LL/DD regime. Two hours after the end of the scotophase, the shelters were carefully removed from the arena and the number of bugs inside each of them was recorded.

Three doses of each of the five compounds (16 ng, 160 ng, and 1.6 µg) were applied in the test shelter in three separate series of assays. These tests were performed separately for *T. infestans*, *P. megistus* and *T. brasiliensis.* Eight replicates were performed for each dose tested with each of the three species. Since our goal was to compare the aggregation inside baited *vs* unbaited shelters, insects found outside refuges were excluded from the statistical analysis because they were not aggregating. It is important to highlight that in triatomines the decision to aggregate or to remain dispersed is influenced by factors such as thigmotaxis and phototaxis, i.e. factors other than the presence of a bait inside a shelter. Therefore, an additional phenomenon would have been evaluated if all insects present in the arena were included in the analysis. The effect of the different doses of compounds on the distribution of insects in baited and unbaited refuges was analyzed by means of a Generalized Linear Model (GLM) using a logistic regression adapted to the binomial nature of the response variable. This analysis was followed by a Wilcoxon signed rank test with continuity correction in order to compare shelter choice data obtained with each concentration and species against a random choice between shelters. Both tests were performed in R software 3.0.2 (R Core Team, 2013).

## Results

### Identification of volatile substances from samples of feces

**Figure 2:**
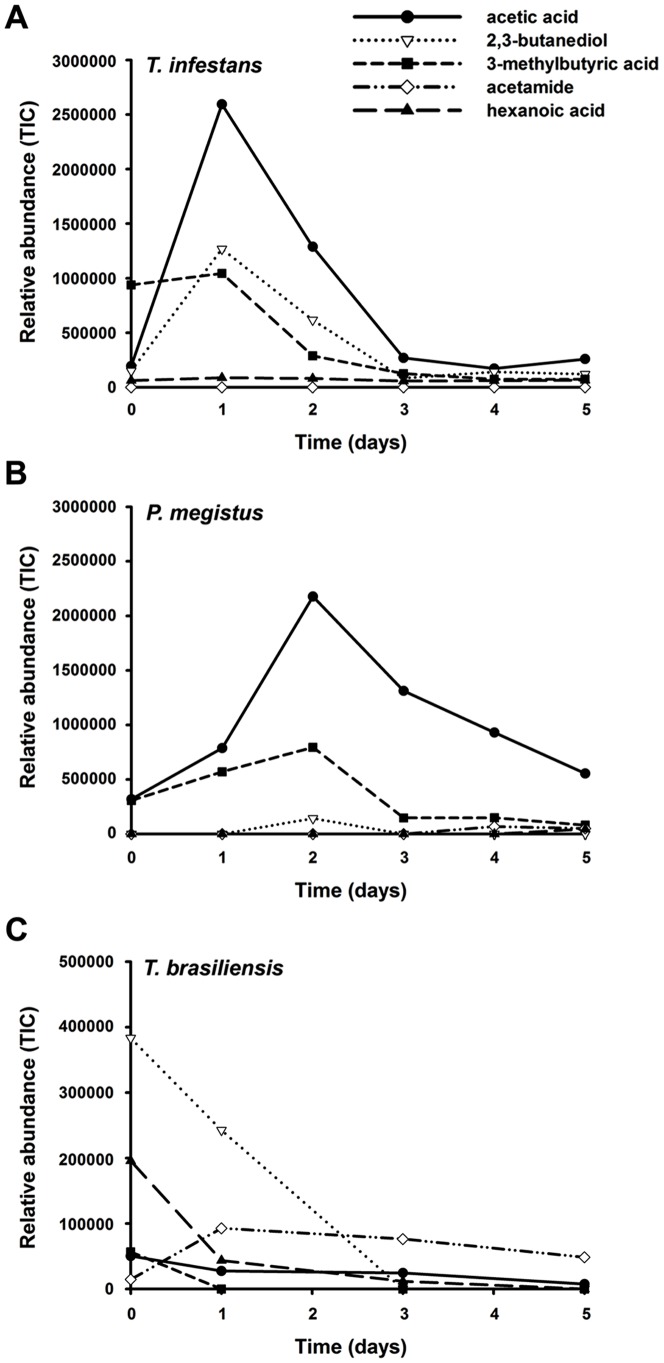
Abundance of odors detected in feces of triatomine bugs. Abundance of acetic acid, 2,3-butanediol, 3-methylbutyric acid, acetamide and hexanoic acid identified in the feces of larvae during five days of sampling. A- *T. infestans*, B- *P. megistus* and C- *T. brasiliensis*. Only traces of acetamide were detected in feces of *T. infestans*.

Five compounds were common to the feces of all species studied: acetamide, 2,3-butanediol, acetic acid, 3-methylbutyric acid and hexanoic acid. The relative abundance and proportion of these five compounds varied during the five days of sampling for the three species: *T. infestans* (Figure 2A), *P. megistus* (Figure 2B) and *T. brasiliensis* (Figure 2C).

In *T. infestans* the abundance of acetic acid and 2,3-butanediol increased markedly 24 h after sample collection before decreasing between the first and second day. 3-Methylbutyric acid was initially present in fresh feces and its abundance decreased until only traces were detectable after 72 h. A relatively constant low abundance of hexanoic acid was detected across the five days of sampling. Only traces of acetamide were detected (Figure 2A).

In samples of feces of *P. megistus* acetic acid was more abundant at the first and second days of sampling, a result which was consistent with our findings in *T. infestans* samples. This was the most abundant compound during all five days. 3-Methylbutyric acid was the second most abundant compound. For *P. megistus*, hexanoic acid, 2,3-butanediol and acetamide were detected as traces (Figure 2B).

In *T. brasiliensis* all compounds were consistently detected in very low amounts over all five days, except 2,3-butanediol, for which the abundance decreased steadily from the first to the fifth day (Figure 2C).

### Behavioral responses to the commonly occurring compounds

Each compound was tested individually for each species. All substances were capable of attracting the three species studied with the exception of 2,3-butanediol which did not induce any effect on *P. megistus* bugs (Table 1). Control tests evaluating two clean filter papers or a solvent control *vs* a clean filter paper presented a random distribution (Binomial test, N.S.).

**Table 1:** Doses of acetamide, 2,3-butanediol, acetic acid, 3-methylbutyric acid and hexanoic acid significantly attractive for *T. infestans*, *P. megistus* and *T. brasiliensis*.

	*T. infestans*	*P. megistus*	*T. brasiliensis*
**Acetic Acid**	100 ng (P<0.009); 100 µg (P<0.02)	10 µg(P<0.004); 100 µg (P<0.008)	10 ng (P<0.01); 100 µg(P<0.005)
**3-Methylbutyric Acid**	10 µg (P<0.009)	100 ng (P<0.01)	100 pg (P<0.03)
**Hexanoic Acid**	100 ng (P<0.02)	1 ng (P<0.03); 10 µg (P<0.01)	10 µg (P<0.01)
**Acetamide**	100 ng (P<0.02)	10 µg (P<0.01); 100 µg (P<0.03)	100 pg (P<0.03); 10 ng (P<0.004)
**2,3-Butanediol**	100 ng (P<0.02); 1 µg (P<0.02)	NS	10 ng (P<0.03)

Results analyzed by means of a Binomial test.

### Blend baited artificial shelters

**Figure 3:**
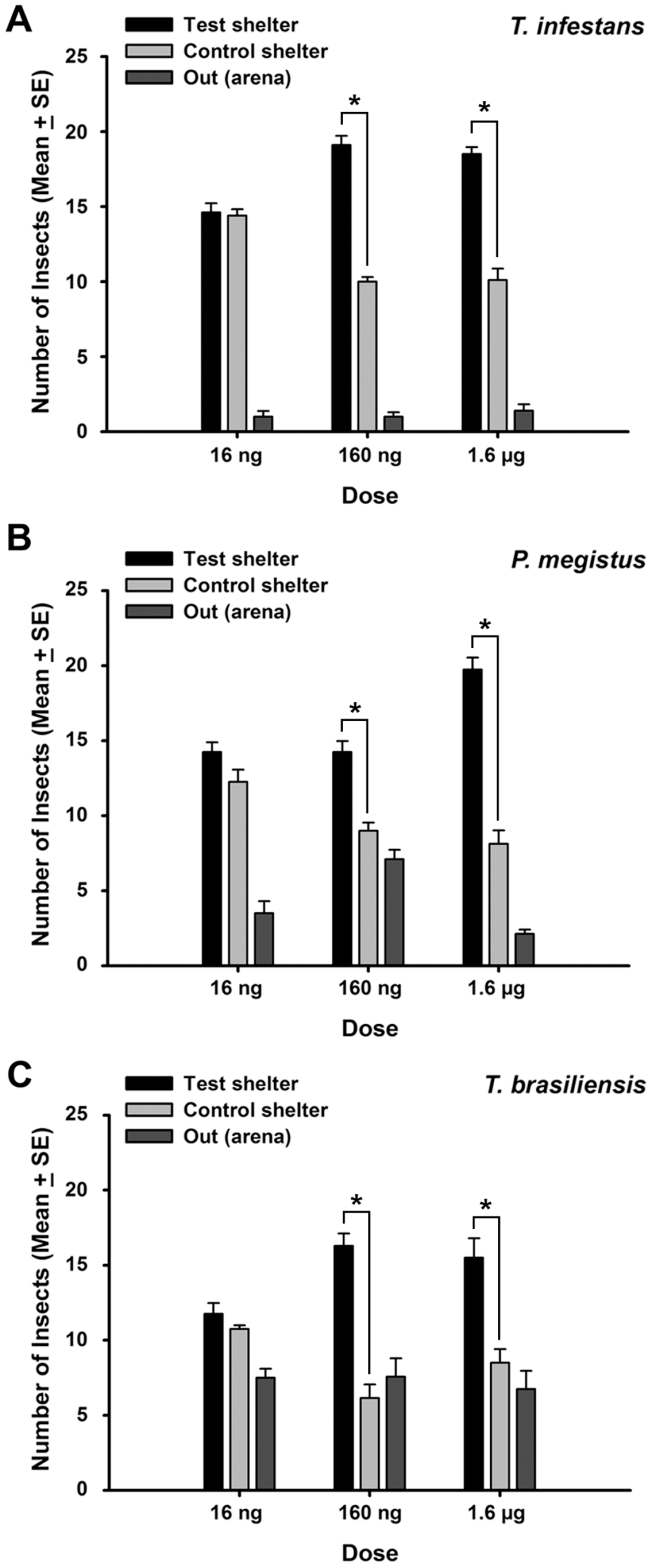
Recruitment of bugs to shelters baited with synthetic blends. Mean number of larvae of A- *T. infestans*, B- *P. megistus* and C- *T. brasiliensis* found inside experimental and control shelters after overnight assays in which mixtures of 16 ng, 160 ng or 1.6 µg of each compound were tested in association with one (test shelter) of two shelters offered. Error bars represent the standard errors of the means. Asterisks indicate significant differences in Wilcoxon signed rank test.

The proportion of insects of each species found inside blend baited and control shelters is presented in Figure 3. The proportion of insects remaining outside refuges at the end of the experiments varied according to the species. The GLM with binomial error revealed a significant effect of the bait dose on the choice for a refuge by the three species (*T. infestans*: z = 3.11, P = 0.00187, residual deviance = 12.940 on 22 degrees of freedom; *T. brasiliensis*: z = 2.403, P = 0.0162, residual deviance = 25.084 on 22 degrees of freedom and *P. megistus*: z = 3.653, P = 0.000259, residual deviance = 13.669 on 22 degrees of freedom). Refuges associated with a mixture of 16 ng of each compound promoted the aggregation of only as many triatomines as clean shelters (Wilcoxon signed rank test, NS, Figure 3). Conversely, shelters associated with mixtures of 160 ng or 1.6 µg of each compound promoted a significantly stronger aggregation on *T. infestans* (160 ng V = 36; P = 0.0078; 1.6 µg, V = 36, P = 0.014), *T. brasiliensis* (160 ng, V = 36, P = 0.0078; 1.6 µg, V = 28, P = 0.022) and *P. megistus* (160 ng, V = 28, P = 0.022; V = 36; P = 0.014) larvae, than clean shelters.

## Discussion

As initially hypothesized, the present study identified volatile compounds common to feces of three species of triatomine vectors. We combined these five substances in a blend that was capable of attracting bugs of the three species into shelters.

In contrast to previous studies we focused on the identification of compounds that were readily available and common to all species. This approach was favored as it is expected to reduce the production cost of chemical baits. We found that the presence of five common compounds was consistent, but their abundance was highly variable throughout the sampling period in all cases. Previously it has been demonstrated that feces, despite this changing proportion of volatiles over time, are attractive for up to 10 days [17]. The low, dynamic abundance and high volatility of the fecal compounds warranted SPME as the sampling method.

Out of the five substances selected for behavioral testing, three have not previously been identified in triatomine feces: 3-methylbutyric acid, hexanoic acid and 2,3-butanediol. Acetic acid and acetamide have been identified in the fecal samples of *T. infestans* and *T. mazzotii* [19], however the biological activity of these compounds was not assessed. The results from our behavioral experiments suggest that they are all constituents of the aggregation signal from triatomine feces and reinforce the hypothesis that this aggregation signal is involved in the marking of shelters by triatomines [7]–[9]. Whether substances other than those reported here play a role in triatomine aggregation remains unclear; nevertheless this is the first report of a chemical blend identified in triatomine feces acting as an aggregation signal for Chagas disease vectors. The results obtained in shelter experiments with all the species studied in this work were very similar, which is consistent with the proposed low specificity of aggregation signals from feces of triatomines [11]–[14], [16]. Therefore, we suggest that our five substance blend could be applied as a general triatomine bait suitable for areas with several sympatric species.

Currently, the detection of domiciliary infestations in control programs is performed by manual search for triatomine bugs and/or colonization signals, such as feces, eggs and exuviae [26]. In cases of low infestation, the use of chemical dislodging agents, e.g., 0.2% tetramethrin, has been introduced to induce insects to abandon their shelters and become exposed [27]. In Argentina, regularly monitored cardboard boxes that offer shelter to bugs in the walls of houses or in their peridomestic structures, have been used [28], [29]. This type of un-baited refuge does not contain glue or insecticide in order to capture or kill visiting insects; instead the bugs find them by chance and generally choose them as a shelter due to their physical properties. The association of these devices with baits, such as the volatile mixture developed in this work, may significantly increase their detection sensitivity. Furthermore, addition of glue [30], insecticide or pathogenic microorganisms [31] may allow transforming these devices into sensitive control tools representing a detection/capture device highly specific for triatomines. One particularly relevant application is the detection of dispersing individuals of sylvatic species that frequently re-invade houses from wild environments after insecticide spraying [32]–[38]. It is important to highlight that under the low density of current infestations in most geographic locations, detection of triatomines is extremely difficult. This limitation may affect the utilization of our mixture that needs to be evaluated under field conditions. We suggest that a long-lasting formulation which would allow a cumulative sampling of bug presence may increase the chances of effective use.

In a broader context, chemical baits based on pheromones or host odors have been proposed as cost effective and environmentally benign alternative tools for detection and control of several pest insects [39], [40], [41]. For triatomines, an odor mixture luring the bugs into detection devices may similarly constitute a practical, economical and environmentally friendly method to monitor infestations by Chagas disease vectors. Further experiments should allow the development of a slow-release formulation for the blend, as well as demonstrate its effectiveness under field conditions. Ultimately, our blend could be developed into more advanced control tools for Chagas disease vectors, which is especially relevant where colonies have developed resistance to current insecticides [30], [31].
